# Topographic controls on soil organic carbon partitioning and enzyme dynamics in nutrient-poor soils

**DOI:** 10.3389/fmicb.2025.1735665

**Published:** 2026-01-13

**Authors:** Yifei Liu, Luge Rong, Yu Cheng, Mingmin Wang, Shuo Min, Fuyou Xiao, Zhiqing Zhang, Ziwei Yang, Qingsong Zhang, Xuehao Zheng

**Affiliations:** 1 School of Geographical Sciences, China West Normal University, Nanchong, China; 2 School of Biomedical and Chemical Engineering, Liaoning Institution of Science and Technology, Benxi, Liaoning, China; 3 Sichuan Provincial Engineering Research Center of Monitoring and Control for Soil Erosion in Dry Valleys, China West Normal University, Nanchong, China

**Keywords:** microbial metabolic shift, sloping farmland, soil enzyme, soil erosion, soil organic carbon, topographic gradient

## Abstract

**Introduction:**

Understanding the dynamics of soil organic carbon (SOC) in sloping farmlands is critical, as they play a vital role in the global carbon cycle and soil health. Although prior research has focused on physical carbon loss due to erosion, the biological mechanisms by which slope gradients affect microbial carbon cycling remain poorly understood.

**Methods:**

Soil samples were collected from maize fields with three slope gradients (30°, 45°, and 60°) across different growth stages. Key indicators were determined as follows: SOC by potassium dichromate oxidation (external heating method); DOC by ultrapure water extraction (1:5 ratio) and organic carbon analyzer; POC by sodium hexametaphosphate dispersion, 53-μm sieving, and chromic acid oxidation; soil Ca^2+^, Mg^2+^, and Cl^−^ by EDTA complexometric titration and silver nitrate titration, respectively; invertase (SUC) by 3,5-dinitrosalicylic acid colorimetry; polyphenol oxidase (SPPO) and peroxidase (SPOD) by commercial kits with L-dopa as substrate. Statistical analyses were performed using IBM SPSS 26 (One-way ANOVA with LSD post-hoc test, Pearson correlation analysis) and Origin 2024 (Principal Component Analysis, PCA). Normality of data was verified prior to analysis, and significance was set at P < 0.05.

**Results:**

Results showed that SOC levels decreased with increasing slope steepness, while DOC peaked at 45°. SPPO and SPOD activities (involved in recalcitrant carbon decomposition) were significantly elevated at 60°. SUC activity was positively correlated with DOC, while oxidase activities were positively associated with POC and negatively with Mg^2+^.

**Discussion:**

This study identifies a critical slope threshold (30°–45°) for DOC loss: DOC availability on steeper slopes stimulates microbial synthesis of SPPO and SPOD, enhancing recalcitrant carbon degradation and potentially intensifying long-term SOC depletion. The identification of this threshold provides insights for designing microbiome-informed strategies to mitigate soil degradation and safeguard ecological security.

## 1 Introduction

Sloping farmlands are critical agricultural resources globally, particularly in mountainous and hilly regions, playing a vital role in ensuring food security and sustaining rural livelihoods (Li and Shi, 2024; Tarolli et al., 2021). Driven by topographic conditions and intense external forces, the farmlands are highly prone to severe soil and water loss, accompanied by land degradation (Sartori et al., 2019). China is one of the world’s most important grain-producing regions. Its sloping farmlands cover approximately 4.867 × 10^5^ km^2^, accounting for over one-third of the total farmland area and supporting the livelihoods of 330 million people in mountainous areas. However, the soil loss rate in these areas exceeds 25 t ha^−1^ yr.^−1^, far surpassing the maximum tolerable soil loss rate (10 Mg ha^−1^ yr.^−1^) (Wang et al., 2024), making sloping farmlands a major source of soil erosion in China (He et al., 2025).

Soil and water loss is influenced by climate, topography, land use, natural disasters, and human activities (Azimi Sardari et al., 2019; Rohit and Villuri, 2025; Santín and Doerr, 2016; Tang et al., 2024), among these, slope gradient is a key topographic factor controlling soil and water loss and soil quality evolution in sloping farmlands. It shortens rainfall infiltration time by enhancing the gravitational component along the slope, significantly increasing runoff velocity and scouring force, thereby driving the process of water erosion and sediment transport (Begum et al., 2010; Parchami-Araghi et al., 2013; Wang et al., 2011). This process disrupts the integrity of biogeochemical cycles in sloping farmlands by stripping away labile soil organic carbon, nutrient ions, and enzyme carriers (Berhe et al., 2018), exacerbating soil function degradation in eroded areas and ecological risks in sedimentation areas (Zhang et al., 2024). Therefore, scientifically clarifying the slope-induced differentiation patterns of labile soil organic carbon, nutrient ions, and enzyme activities in sloping farmlands is of great significance for understanding erosion processes, assessing carbon loss risks, and formulating soil and water conservation strategies.

The Liangshan Yi Autonomous Prefecture is located in the arid and semi-arid regions of southwestern China. It serves as an important grain production base in southwestern China and is also an area prone to frequent soil and water loss. Areas with a slope gradient of less than 5° account for only approximately 5.21% of the total area, while areas with a slope gradient greater than 15° exceed 65%. Thus, compared with other regions, it relies much more heavily on agriculture on steep slopes (Ni et al., 2016). Under natural conditions, maize is the dominant crop in the local sloping farmlands (Wang et al., 2019). Several studies have shown that vegetation plays a crucial role in soil and water conservation in sloping farmlands. For example, vegetation can directly cover the soil surface through canopies or litter layers to prevent raindrop erosion, thereby reducing soil and water loss in the short term (Lacombe et al., 2018), affect soil carbon dynamics through the input of root residues or rhizodeposits at different growth stages (Pehlivan et al., 2025; Qiao et al., 2017). Vegetation can also influence soil enzyme activity by secreting chemical substances into the rhizosphere and reacting with acids in the soil, thereby altering the soil pH (Kaźmierczak et al., 2025). In this region, the concentrated rainfall period (July–September) overlaps temporally with the peak vegetation growth season, significantly increasing soil erosion intensity (Li et al., 2024), and existing studies have shown that slope gradient has a significant impact on the soil and water conservation performance of vegetation (Ochoa et al., 2016). Therefore, due to the dynamic fluctuations in vegetation cover with plant growth stages (Li et al., 2024), vegitation-driven chemical changes may interact intricately with slope-induced physical erosion, together shaping the spatiotemporal patterns of labile soil organic carbon, ion balance, and enzyme activity in sloping cropland systems. Additionally, most existing studies have focused on gentle or moderately sloped croplands (<25°), while systematic research on soil health indicators under steep slope cultivation conditions (e.g., >25°) remains relatively scarce. In reality, such steep sloping croplands are still widely distributed in Liangshan Prefecture and pose extremely high ecological risks. The lack of process-based research on soil health indicators in steep sloping farmlands limits our scientific understanding of their degradation patterns and effective prevention and control measures.

This study aimed to (1) determine the dynamics of soil organic carbon (SOC) fractions, (2) assess changes in soil ion content, (3) detect soil enzyme activities, and (4) analyze the relationships among soil active organic carbon fractions, soil ions, and enzyme activities. We hypothesized that the steepest slope (60°) would exhibit lower SOC fraction contents compared to the other two slopes, as steeper slopes are associated with more severe soil and water erosion. We further hypothesized that the dynamics of these erosion-driven SOC fractions would be associated with variations in enzyme activities, as the biogeochemical processes of the carbon cycle, which are central to regulating SOC fraction transformations, are primarily mediated by microbially produced enzymes. The findings of this study are expected to provide scientific insights for risk assessment, optimization of soil and water conservation measures, and adaptive soil management of steep sloping farmland (especially >30°), thereby helping to curb farmland degradation and safeguard regional food security and ecological security.

## 2 Materials and methods

### 2.1 Study area description

**Figure 1:**
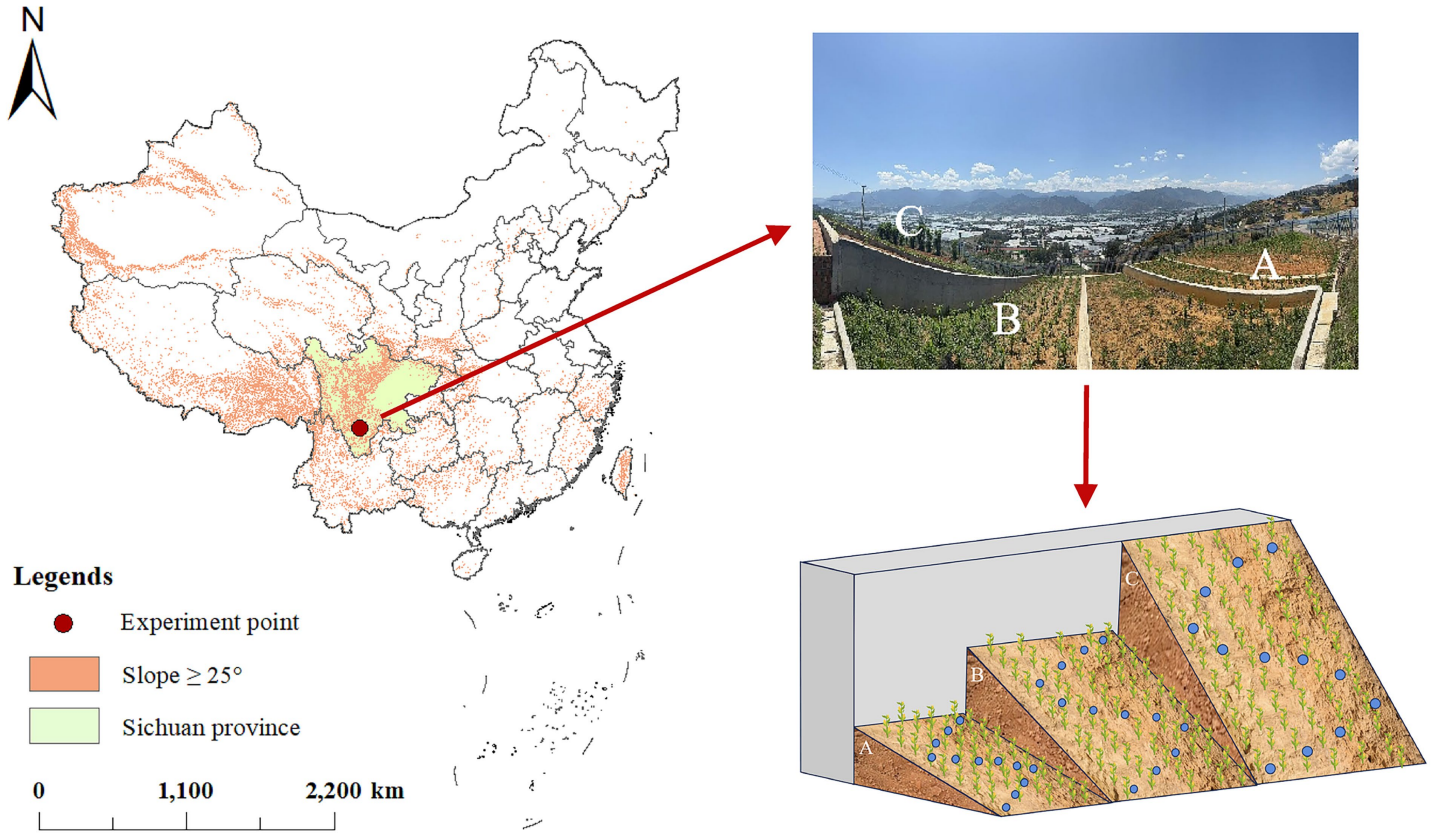
Location, slope distribution, and the experimental field.

The study site is located in Xide County (27°53′–28°31′N, 102°12′–102°43′E), Liangshan Yi Autonomous Prefecture, Sichuan Province, China (Figure 1). This region experiences a Subtropical Monsoon Climate with low latitude and high altitude. It lies on the eastern flank of the Hengduan Mountains, in the southwestern part of the Sichuan Basin, characterized by a deeply incised valley and low hilly topography, with an average elevation of approximately 1700 m. Cultivated land make up only 10.6% of the total area in Xide County, and within this limited portion, over 60% lies on slopes steeper than 25° (Luo et al., 2023). The overall quality of this farmland is generally poor, with an average yield of merely 284 kg per mu, representing only about 76.78% of the national average (Liu et al., 2011). The primary crops cultivated on these farmlands are wheat (*Triticum aestivum* L.), maize (*Zea mays* L.), and sweet potato [*Ipomoea batatas* (L.) Lam.]. The predominant soil type is mountain yellow-brown earth, which has developed from Quaternary Loose Sediments. This soil is characterized by suboptimal structure, high sand and gravel content, and limited capacity for retaining water and nutrients. Due to active tectonic forces, complex topography, and severe fluvial erosion (Xu et al., 2024), the area is highly susceptible to soil and water loss.

### 2.2 Sampling plot establishment and soil sample collection

The experimental runoff plots measured 4.0 m in length by 2.0 m in width. Based on the typical topography and slope distribution characteristics of Xide County, three slope gradients were established: 30° (A), 45° (B), and 60° (C) (Figure 1). Each slope gradient had one replicate. Within each plot, 12 sampling points were uniformly arranged along an “S”-shaped pattern with a spacing of approximately 1.4 meters between points. Soil samples collected from these 12 points were thoroughly mixed to form a composite sample per plot, and no additional plot replicates were established. During sampling, care was taken to avoid maize roots. The maize cultivar used in the experiment was “Liangdan 6”, a dominant local cultivar widely grown in Xide County, sown on April 25, 2024. Field management and fertilization practices followed local conventions throughout the entire growth cycle, while fertilization was applied once before sowing with no topdressing thereafter, which was intended to eliminate interference from exogenous carbon inputs on soil organic carbon fractions.

Soil samples from the 0–20 cm surface layer, which is the primary zone of maize root activity and organic carbon accumulation, were collected during three key growth stages: Vegetative Emergence (VE, May 2), Vegetative Tasseling (VT, July 18), and Physiological Maturity (PM, October 2). A stainless-steel shovel was used for collection. 12 soil samples collected from each plot were thoroughly mixed, then reduced in volume using the quartering method to retain a representative subsample. After removing impurities, the subsample was placed in a sealed plastic bag and promptly transported to the laboratory. The soil sample was naturally air-dried in a well-ventilated and cool area. Subsequently, the soil was ground and sieved through a 0.4-mm mesh sieve (for enzyme activity) and a 2-mm mesh sieve (for SOC, labile carbon fraction analysis and ion analysis) to prepare for experimental analysis and determination.

### 2.3 Soil analysis methods

SOC was determined using the potassium dichromate oxidation method with external heating. DOC was extracted with ultrapure water at a 1:5 ratio and measured with an organic carbon analyzer (multi-N/C 3100, Analytik Jena, Germany). Soil readily oxidizable organic carbon (EOC) was determined using the potassium permanganate-UV–visible spectrophotometry method. Particulate organic carbon (POC) was isolated using sodium hexametaphosphate dispersion and sieving through a 53-μm sieve. The soil retained on the sieve was repeatedly washed with distilled water, dried at 60 °C, weighed, and measured using the chromic acid oxidation method (Liao et al., 2023). Water-soluble soil calcium (Ca^2+^) and magnesium (Mg^2+^) ions were measured by EDTA complexometric titration. Chloride ion (Cl^−^) content was determined by silver nitrate titration. SUC activity was assayed using the 3,5-dinitrosalicylic acid colorimetric method and expressed as the mass of glucose (mg) produced per gram of soil per hour after a 24-h incubation at 25 °C (Li et al., 2025). Soil polyphenol oxidase (SPPO), peroxidase (SPOD), and neutral phosphatase (SNP) activities were determined using commercial assay kits (provided by Suzhou Geruisi Biological Technology Co., Ltd., China). Specifically, both SPPO and SPOD were assayed with L-dopa as the substrate, while SNP used p-nitrophenyl phosphate as the substrate. And all assays were incubated for 1 h. Enzyme activities were expressed as the amount (nmol) of red product (for SPPO and SPOD) or p-nitrophenol (for SNP) produced per gram of soil per hour during incubation at 25 °C.

### 2.4 Statistical analysis

Data analysis was performed using IBM SPSS Statistics software (Version 26). Prior to statistical analysis, the normality of the data was assessed using the Kolmogorov–Smirnov test, data that did not follow a normal distribution were subjected to appropriate transformation. One-way analysis of variance (One-way ANOVA) was employed to compare the differences in soil organic carbon fractions, ion contents, and enzyme activities across different slope gradients and sampling periods. Post-hoc multiple comparisons were conducted using the Least Significant Difference (LSD) method. Pearson correlation analysis was used to examine the relationships between soil ions, enzyme activities and organic carbon components. Principal Component Analysis (PCA) was performed and visualized using Origin software (2024). For the PCA, variables with communalities of less than 0.5 were excluded, and principal components were selected based on the latent root criterion (eigenvalues greater than 1.0). Data are presented as the mean ± standard deviation. The significance level was set at *p* < 0.05 for all statistical tests.

## 3 Results

### 3.1 Dynamics of soil labile organic carbon fractions on different slopes during maize growth

**Figure 2:**
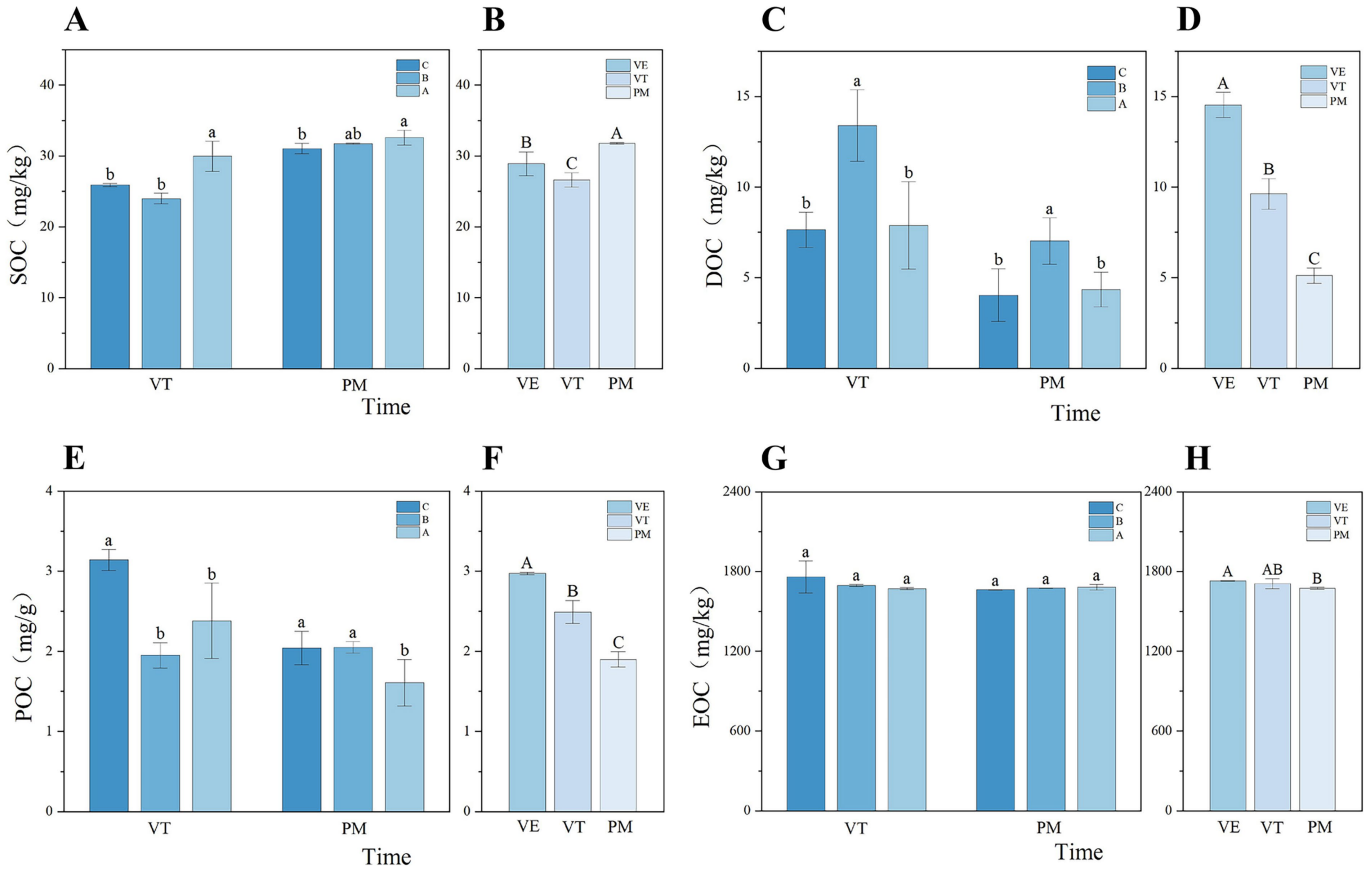
Dynamic variation characteristics of labile soil organic carbon content under different slope gradients **(A,C,E,G)** during maize growth stages **(B,D,F,H)**. A represents 30°, B represents 45°, and C represents 60°; VE represents vegetative emergence stage, VT represents vegetative tasseling stage, and PM represents physiological maturity. Lowercase letters indicate significant differences between slope gradients within the same growth stage, while uppercase letters indicate significant differences between growth stages within the same slope (*p* < 0.05). Error bars indicate standard deviation.

One-way analysis of variance was performed on the measured data of soil organic carbon and its fractions to determine the significance of differences among the various slope gradients and to further investigate the influencing factors behind these observed patterns. At the VT stage, SOC content exhibited an initial decrease followed by an increase with increasing slope gradient, with the content at Slope A (30°) being significantly higher than that at Slope B (45°). In contrast, during the PM stage, SOC content decreased significantly with increasing slope gradient (Figure 2A). For DOC, the trends in response to increasing slope gradient were remarkably similar at both the VT and VE stages: content initially increased, peaked at Slope B, and then decreased, with the values at the other two slopes being significantly lower than that at Slope B (Figure 2C). In comparison, the differences in EOC content across different slope gradients were not significant at any of the sampling periods (Figures 2G,H).

Overall, across the three distinct growth stages of maize, the contents of soil organic carbon and its fractions generally showed a declining trend as the plant developed (Figure 2E). The decreasing trend was most pronounced for DOC, followed by POC (Figures 2D,F). It is noteworthy, however, that SOC content at the PM stage showed a rebound and was significantly higher than that during the other two growth stages (Figure 2B).

### 3.2 Dynamics of soil soluble ion contents on different slopes during maize growth

**Figure 3:**
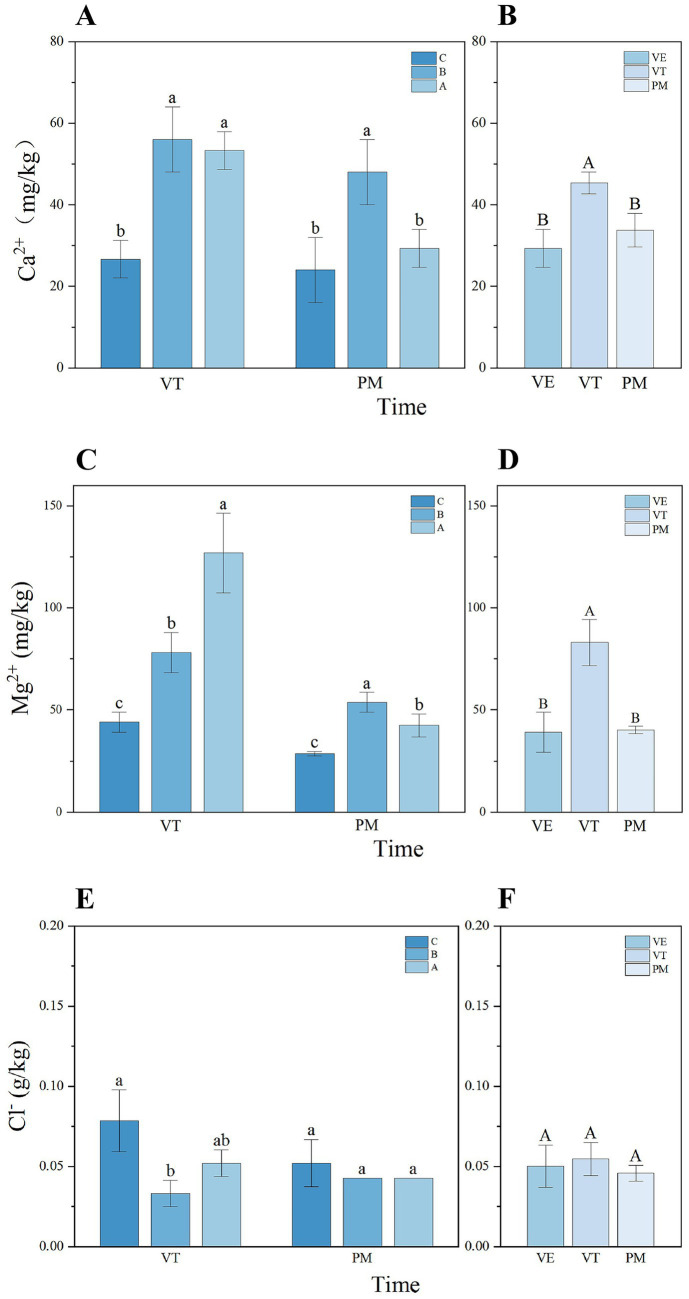
Dynamic variation characteristics of soil ion content under different slope gradients **(A,C,E)** during maize growth stages **(B,D,F)**. Lowercase letters indicate significant differences between slope gradients within the same growth stage, while uppercase letters indicate significant differences between growth stages within the same slope (*p* < 0.05). Error bars indicate standard deviation.

In sloping farmland, soil ions not only supply essential nutrients for plant growth but also play a key role in maintaining soil structural stability and environmental health. This study analyzed the contents of Ca^2+^, Mg^2+^, and Cl^−^ in the soil. During the VE stage, Cl^−^ content exhibited an initial decrease followed by an increase with rising slope gradient, reaching its maximum value at Slope C (Figure 3E). In contrast, the variation trend of Ca^2+^ content was opposite, with its value at Slope C being significantly lower than those at the other two slopes (Figure 3A). At the PM stage, the contents of Mg^2+^ and Ca^2+^ showed an inverted “U”-shaped distribution across slopes, peaking significantly at the intermediate Slope B, while no significant topographic differentiation was observed for Cl^−^ content during this period (Figure 3C).

In general, across the three maize growth stages, the contents of Ca^2+^, Mg^2+^, and Cl^−^ all showed distinct temporal dynamics (Figures 3B,D,F). Specifically, Cl^−^ content was highest at the VE stage and decreased during the VT and PM stages. The contents of Ca^2+^ and Mg^2+^ were relatively high at the VT stage but declined by the PM stage, although Ca^2+^ still maintained a relatively high level at PM. These dynamics are likely driven by the shifting nutrient demands during maize growth and the concurrent changes in soil nutrient availability.

### 3.3 Dynamics of soil enzyme activities on different slopes during maize growth

**Figure 4:**
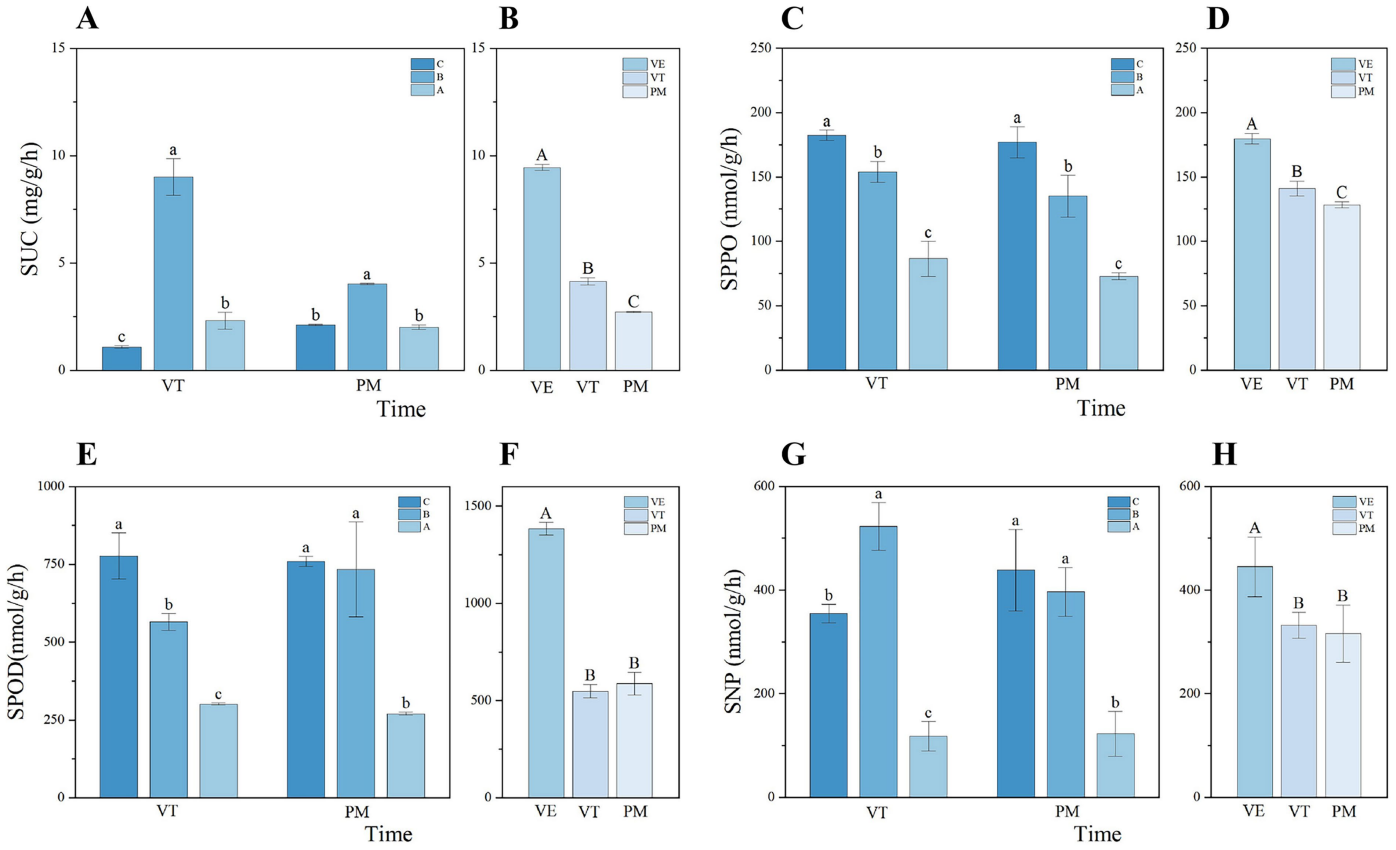
Dynamic variation characteristics of soil enzyme activities under different slope gradients **(A,C,E,G)** during maize growth stages **(B,D,F,H)**. Lowercase letters indicate significant differences between slope gradients within the same growth stage, while uppercase letters indicate significant differences between growth stages within the same slope (*p* < 0.05). Error bars indicate standard deviation.

Similar to soil organic carbon, enzymes serve as critical indicators of specific soil functions (Bagavthsingh and Duraisamy, 2024). At the VT stage, SUC and SNP activities showed a distinct increase followed by a decline with rising slope gradient. With an increase of over 50%, enzymatic activities at Slope B were significantly higher than those at the other slopes A and C (Figures 4A,G). The topographic variations in SPPO and SPOD activities during the VT stage were similar, showing a consistent upward trend with increasing slope steepness. This pattern was also observed for SPPO, SPOD, and SNP activities during the PM stage (Figures 4C,E,G), with peak values recorded at the steepest slope (Slope C). In contrast, SUC activity exhibited an inverted “V”-shaped distribution across slopes during this period, with significantly higher activity at the intermediate slope compared to the other two gradients. Overall, soil enzyme activities declined progressively across the three maize growth stages, reflecting a general reduction in microbial functional intensity as the crop matured (Figures 4B,D,F,H).

### 3.4 Correlations of soil enzyme activities with soil total organic carbon and its labile fractions

**Figure 5:**
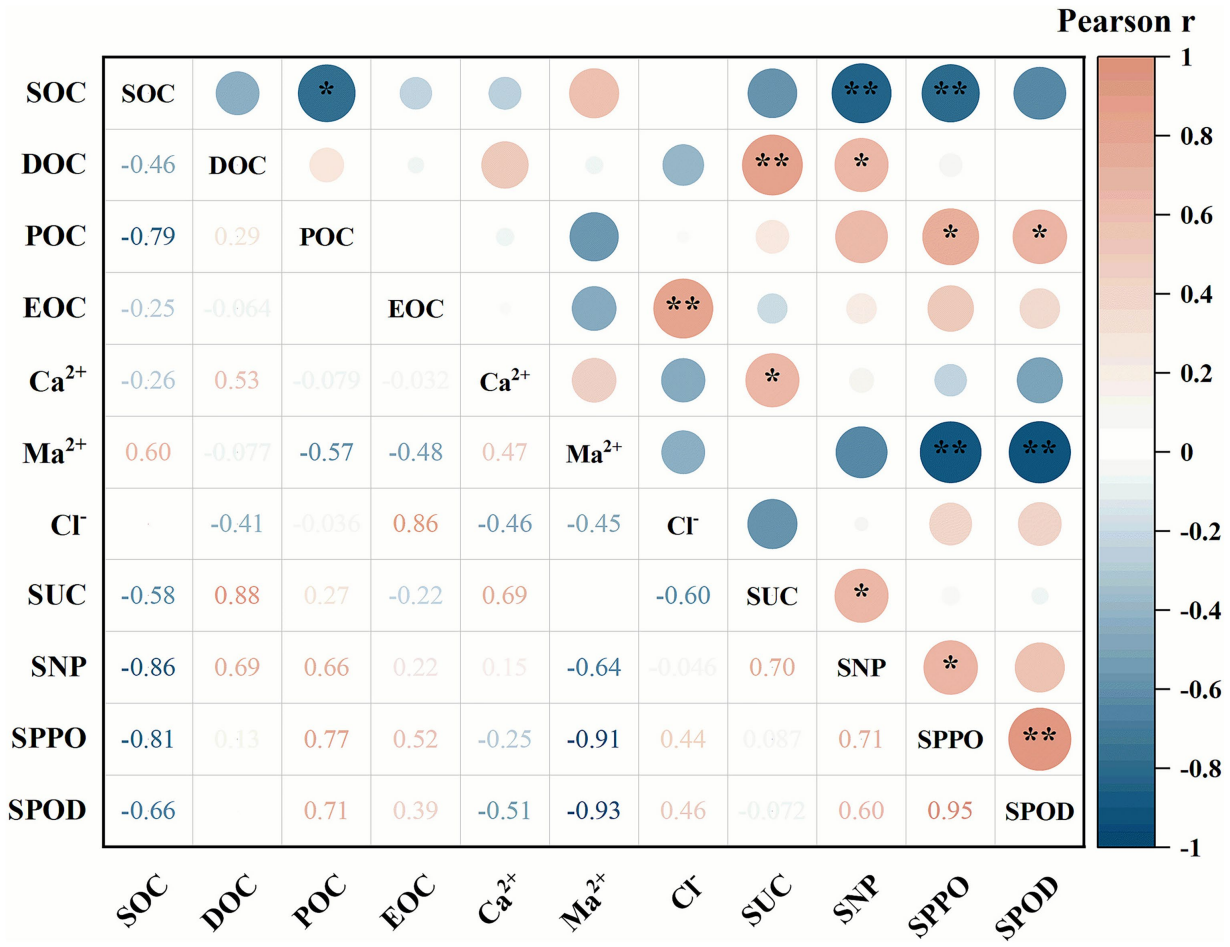
Correlations of the four soil enzyme activities with soil ion content and soil labile organic carbon fraction contents. *, *p* < 0.05; **, *p* < 0.01.

Pearson correlation analysis was employed to assess the relationships between the metrics. The analysis revealed that soil enzyme activities of SUC, SNP, SPPO, and SPOD exhibited significant correlations with several soil organic carbon fractions and ions (Figure 5). Specifically, SUC activity showed a strong positive correlation with DOC content, but no significant correlation with SOC, EOC, or POC. However, SUC activity also correlated positively with Ca^2+^ content, suggesting a potential role of calcium in stabilizing enzyme function. SNP activity demonstrated a strong negative correlation with SOC content, coupled with a significant positive correlation with DOC. SPPO activity was significantly negatively correlated with SOC and Mg^2+^ levels, while showing a significant positive correlation with POC content and SNP activity. Finally, SPOD activity exhibited a significant positive correlation with POC, a very strong positive correlation with SPPO, and a highly significant negative correlation with Mg^2+^ content.

### 3.5 Principal component analysis

Principal component analysis (PCA) is a powerful statistical method used to condense the information from a large, complex dataset into a smaller, more manageable set of components for easier visualization and interpretation. PCA was performed based on the measured active organic carbon fractions, soil enzyme activities, and soil ions across different slope gradients and growth stages to identify the main sources of environmental variation. The results indicated that the first three principal components (PCs) collectively accounted for 77.365% of the total variance, suggesting that these PCs successfully captured the majority of the information from the 12 environmental factors. The first principal component (PC1), which explained 34.653% of the variance, was primarily driven by total SOC, with a loading of 0.404. The second principal component (PC2), accounting for 26.458% of the variance, was dominated by Ca^2+^ and Mg^2+^, with loadings of 0.430 and 0.418, respectively. Finally, the third principal component (PC3), explaining 16.254% of the variance, was mainly associated with Cl^−^, which had a loading of 0.424 (Table 1).

**Table 1:** Principal component analysis of slope gradients and maize growth stages based on soil physical and chemical properties and soil enzyme activities.

Factors	Principal Components		
	1	2	3
pH	−0.274	0.262	−0.235
SOC	−0.404	0.139	−0.198
DOC	0.363	−0.268	−0.042
POC	0.256	0.243	0.429
EOC	0.211	0.144	0.290
Ca^2+^	0.120	−0.430	0.093
Mg^2+^	−0.016	−0.418	0.392
Cl^−^	0.105	0.375	0.424
SUC	0.305	−0.300	−0.324
SNP	0.380	0.064	−0.357
SPPO	0.388	0.274	−0.114
SPOD	0.325	0.304	−0.219
Eigenvalue	1.757	0.388	0.141
Percent (%)	34.653	26.458	16.254
Cumulative percent (%)	34.653	61.111	77.365

**Figure 6:**
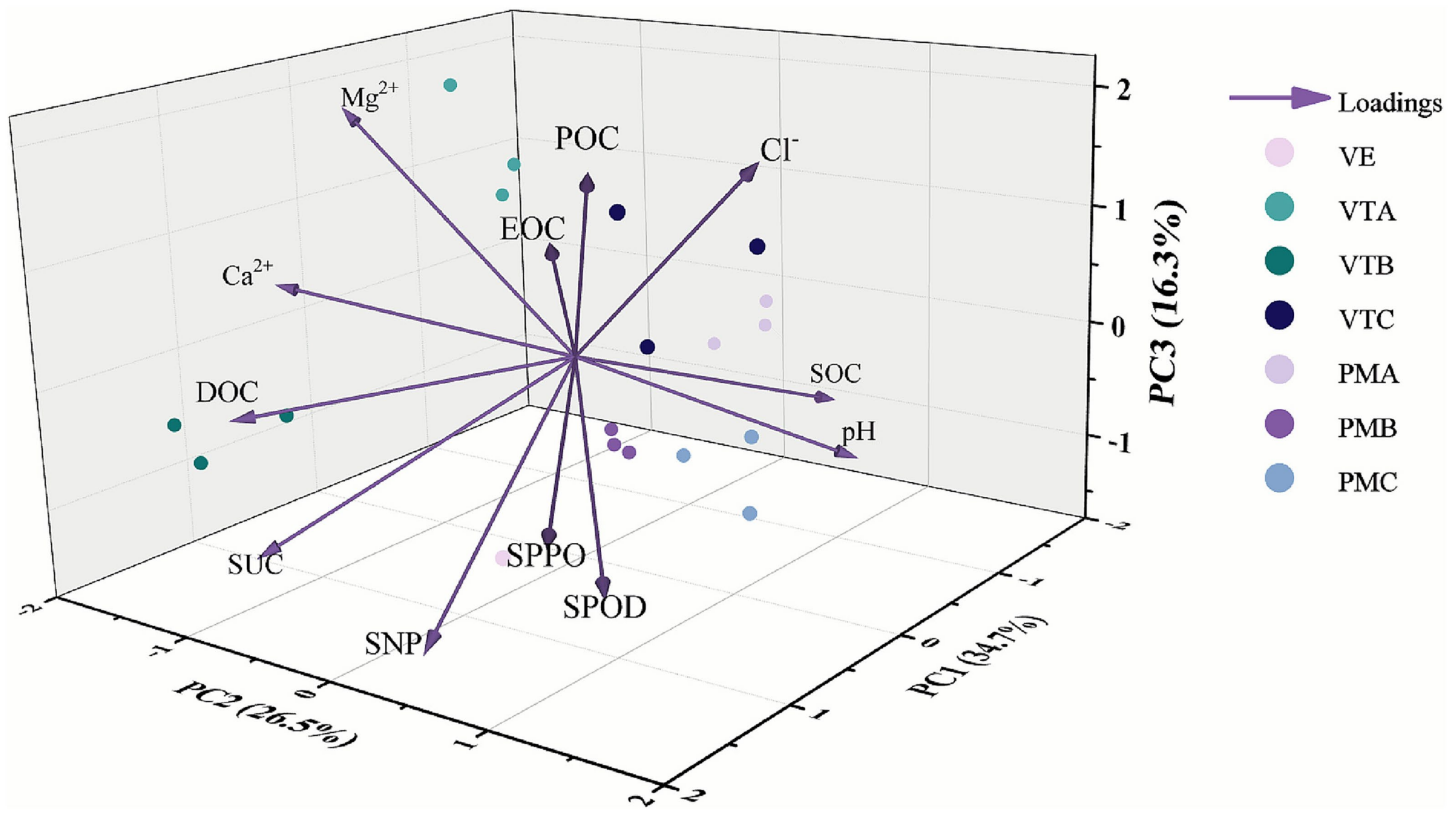
Principal component analysis ranking chart. VTA, VTB, and VTC represent 30°, 45°, and 45° slope during vegetative tasseling stage; PMA, PMB, and PMC represent 30°, 45°, and 45° slope during physiological maturity.

The PCA results (Figure 6) further indicated that the HDC sampling points were positioned close to the vectors for SUC and DOC, suggesting that the 30° slope had a discernible influence on both SUC activity and DOC content during the maize maturation stage. Similarly, the HDA sampling sites were located near the vectors for POC and EOC, implying a significant effect of the 60° slope on these two labile organic carbon fractions at maturity. The PMC points clustered near the SOC vector, indicating a notable impact of the 30° slope on soil organic carbon during the maize heading stage. Furthermore, the VE samples were associated with the vectors for SPOD and SPPO, demonstrating that the seedling stage exerted a significant influence on the activities of these two enzymes. Other data points were more dispersed within the ordination space and did not show clear proximity to any specific soil enzyme activity vector. The biplot also revealed that the vectors for Mg^2+^, Ca^2+^, and DOC were relatively long and formed acute angles with each other, denoting marked positive correlations among these variables. The extended vector for DOC and its acute angles with the vectors for SNP and SUC further indicated positive correlations between DOC content and the activities of these two enzymes.

## 4 Discussion

### 4.1 Response of soil total organic carbon and its labile fractions to different slope gradients

SOC plays a central role in maintaining soil fertility and contributes significantly to soil development and functional enhancement (Zhao et al., 2023). This study demonstrates that slope gradient, as an environmental factor, significantly influences SOC and its labile fractions across different maize growth stages. Overall, SOC exhibited a declining trend with increasing slope steepness. Specifically, during the VT and PM stages, the SOC content on the 60° slope was significantly lower than that on the 30° slope (Figure 2A). These results support our first hypothesis. This trend may result from accelerated surface runoff on steeper slopes, which enhances its detaching capacity, leading to the loss of larger soil particles and aggregates, thereby exacerbating the transport and depletion of organic carbon (Hou et al., 2020; Niu et al., 2024; Shi et al., 2019).

In contrast, the dynamics of labile organic carbon fractions differed from those of total SOC. During the VT and VE stages, DOC content peaked at the 45° slope, showing significantly higher values compared to the other gradients (*p* < 0.05) (Figure 2B), indicating a non-linear response. This phenomenon may be governed by the interplay between micro-topography and the critical slope gradient. Liu et al. (2001) propose a universally applicable critical slope range of 41.5°–50° for soil erosion through theoretical derivation and numerical simulations, which is independent of regional-specific conditions. Our study, conducted in Xide County’s mountainous sloping farmland with distinct local soil properties and geomorphology identified a 45° critical slope that falls within this validated range, and this slope coincided with the peak in DOC content. The consistency of results across different regional contexts directly verifies the cross-regional applicability of the critical slope dynamic, further supporting our conclusion. Within this range, there is a positive correlation between slope gradient and soil erosion; beyond this threshold, the relationship shifts to negative. The results of this study also confirm this conclusion. The reason for the peak DOC content at the 45° slope may be that DOC tends to migrate at the soil-water interface with runoff (He et al., 2023). During the study period, rainfall data for Xide County retrieved from the official website of the National Meteorological Centre (NMC) show a total rainfall of 680–840 mm, which is sufficient to induce slope runoff. Rainfall erosion at this slope range further leads to the formation of rills, which increases water retention and infiltration (Kayombo et al., 1991; Wang et al., 2017), thereby reducing runoff-driven DOC loss. However, when the slope gradient exceeds the critical threshold, the interaction between hydraulic erosion and gravitational erosion is significantly enhanced (Cui et al., 2024), which greatly improves sediment transport capacity and causes a surge in DOC migration, ultimately resulting in a decrease in DOC content. Therefore, identifying the critical slope gradient is of great significance for soil carbon management.

SOC fractions also exhibited differences in variation across different maize growth stages (He et al., 2023). From the VE to the PM stage, the contents of DOC and POC decreased significantly, while SOC showed a significant increasing trend at the maturity stage (*p* < 0.05) (Figures 2D,F). In contrast to earlier findings (Qi et al., 2016), labile organic carbon fractions did not increase significantly with SOC due to high microbial activity during vigorous crop growth. Labile organic carbon fractions are utilized by microorganisms and converted into microbial-derived carbon compounds (a secondary source) and CO₂ (Zhang et al., 2023), leading to a measurable decline in labile carbon levels. The variation in SOC is essentially the result of a dynamic balance between carbon input and output. During the maize seedling and heading stages, the amount of carbon consumed by microorganisms was greater than that converted. However, at the maturity stage, maize inputs a large amount of organic carbon into the soil through its well-developed root system. On one hand, this carbon is consumed by microorganisms (Liu et al., 2014), which maintains POC and DOC at relatively low levels. On the other hand, through the “microbial carbon pump” effect, plant residues are transformed into more stable recalcitrant organic carbon via extracellular modification and intracellular turnover (Cotrufo et al., 2015; Liang et al., 2017). Therefore, the net carbon input exceeded the net output, leading to an increase in the total SOC content. The divergence in variation trends between POC, DOC, and SOC further indicates that root carbon input plays a crucial role in regulating SOC changes during the growing season. These carbon fraction dynamics are closely linked to soil enzyme activities, as soil enzymes respond to substrate availability and biogeochemical demands; thus, their responses to different slope gradients may further reflect how these carbon fraction variations drive changes in soil enzyme activities.

### 4.2 Response of soil enzyme activities to different slope gradients

Soil enzyme activity is a fundamental driver of soil biochemical processes (Wang et al., 2023; Cheng et al., 2025). Due to its high environmental sensitivity, it serves as a critical biological indicator for assessing soil quality and health status (Wu et al., 2019). Topographic variation influences enzyme activity by altering litter input, root exudation, and microclimatic conditions (Qu et al., 2025). Soil enzymes decompose complex organic compounds, providing energy for soil microorganisms (Gao et al., 2021). Prior study (Mandal et al., 2021) suggested that with increasing slope gradient, intensified soil erosion washes away soil organic carbon and fine particles which act as enzyme carriers and substrates, thereby reducing soil enzyme activity. However, our finding revealed a contrasting pattern, where SPPO and SPOD activities increased progressively with slope gradient, challenging the conventional understanding. This discrepancy indicates that the response of soil enzyme activity to topographic factors is not strictly linear but is modulated by both enzyme functional types and substrate availability.

As key enzymes for degrading lignin and other recalcitrant organics (Li et al., 2022), increased SPPO and SPOD activity likely reflects enrichment of lignin-degrading microorganisms in response to labile carbon limitation (Figure 2C). This supports the Resource Allocation Theory (Wutzler et al., 2017), which posits that microorganisms prioritize synthesizing enzymes targeting recalcitrant substrates when labile carbon sources are scarce, to maintain metabolic activity. Lignin contrasts with labile organics that microorganisms can directly utilize, while DOC is mainly composed of these soluble and labile substances (Aumtong et al., 2023). With increasing slope gradient, DOC content decreases, reducing soil labile carbon availability. Under this limitation, microorganisms may shift to utilizing complex organics, thereby stimulating the synthesis and activity of these oxidase systems. This result preliminarily indicates that the impact of topography on soil carbon turnover is not limited to the loss of physical carbon pools. It may also profoundly regulate microbial metabolic pathways by altering carbon source availability. Therefore, future assessments of sloped ecosystems should go beyond mere changes in carbon storage. They should further explore the adaptive responses of microbial communities and their ecological effects under resource stress. This will help reveal the dynamic process of soil organic matter decomposition and its ecological significance more accurately.

### 4.3 Relationships among soil enzyme activities, labile organic carbon, and ions

The research findings indicate that there exists a complex and functionally distinct interaction between soil enzyme activities and key physicochemical properties, particularly specific soil labile organic carbon fractions and specific cation concentrations (Figure 5). This interaction is further significantly modulated by spatiotemporal factors, including maize growth stages and slope gradients (Figure 6). Specifically, SUC activity exhibits an extremely significant positive correlation with DOC content, while showing no significant associations with SOC or other labile carbon pools. This observation indicates that SUC activity is primarily fueled by the most readily bioavailable carbon sources in soil (Song et al., 2019). Additionally, SUC activity correlates significantly positively with Ca^2+^, suggesting that Ca^2+^ may exert a stabilizing or enhancing effect on this enzyme activity by reducing enzyme denaturation in nutrient-poor soils, which is consistent with previous observations regarding cation-enzyme activity interactions (Kravkaz Kuscu et al., 2018). In contrast, SNP activity presents a more complex correlational pattern. Its positive relationship with DOC content and negative relationship with SOC content are supported by PCA. This phenomenon implies that phosphorus acquisition processes are not merely constrained by total carbon content; rather, they may be stimulated in environments enriched with labile carbon (e.g., DOC). This finding further validates the “microbial nutrient mining” mechanism (Keiluweit et al., 2015; Xiao et al., 2021), simultaneously reinforcing the conclusion that DOC functions as a key trigger for hydrolase activity (Bai et al., 2024). The negative correlation between SOC content and SNP activity may reflect an alternative scenario: high SOC levels in soil that is potentially in more recalcitrant forms could coincide with phosphorus accumulation or shifts in microbial community composition, thereby reducing the requirement for acid phosphatase biosynthesis.

As the primary enzymes responsible for degrading recalcitrant aromatic compounds, oxidases (SPPO, SPOD) have correlational patterns that are distinctly different from those of hydrolases (SUC, SNP). Notably, both SPPO and SPOD activities display an extremely significant negative correlation with Mg^2+^ content. This negative association suggests that Mg^2+^ may indirectly inhibit oxidase activity by suppressing the metabolic activity of enzyme-producing microorganisms, impairing the catalytic activity of the enzymes themselves, altering the soil physicochemical microenvironment (Baldrian, 2003; Mikutta et al., 2007; Sinsabaugh, 2010) or influencing organo-mineral associations to reduce the physical accessibility of substrates to enzymes—a mechanism supported by evidence that organo-mineral interactions can modulate substrate availability for soil enzymes (Mikutta et al., 2007). Additionally, SPPO activity shows an extremely significant negative correlation with SOC. This may indicate that phenol oxidase activity is repressed in soils with high stable organic matter content, or conversely, that such enzymes are only synthesized in large quantities when microorganisms need to target the decomposition of recalcitrant carbon pools, which does not always align with positive correlations with total SOC content.

Of particular note, SPOD activity not only correlates significantly positively with POC content but also exhibits an extremely significant positive correlation with SPPO activity, indicating a potential synergistic relationship between the two enzymes (Figure 5). PCA analysis further reveals that the slope gradient (HDA) variable clusters closely with the POC vector (Figure 6). This spatial distribution suggests that soil environments on steep slopes may be more favorable for POC accumulation or exposure, thereby specifically activating the oxidase system dominated by SPPO and SPOD and further facilitating the decomposition of complex organic substances. This result confirms the hypothesis proposed in our earlier discussion. As a partially degraded yet particulate organic carbon fraction, POC appears to be another key substrate that triggers the production of the oxidase system. The synergistic effect between SPPO and SPOD further validates their classical cooperative mechanism in degrading phenolic polymers (e.g., lignin) (Hammel and Cullen, 2008; Sinsabaugh, 2010), which is a critical rate-limiting step in the soil carbon cycle. Collectively, these results emphasize that microbial community-mediated decomposition processes are not solely regulated by total soil organic carbon content. Instead, they are finely adjusted by the bioavailability of specific carbon sources and the soil cationic environment. Therefore, future evaluations of soil carbon cycles should transcend a sole focus on total organic carbon content and instead prioritize investigating the interactions between specific carbon fractions, key soil cations, and the diverse functional enzymes that drive decomposition processes.

## 5 Conclusion

This study showed that DOC content peaked at a critical slope gradient of ~45°, whereas oxidase (SPPO, SPOD) activities were more pronounced on steeper slopes. This finding reveals a key mechanism for the shift in microbial metabolic pathways from labile carbon utilization to recalcitrant carbon-degrading oxidase production. Correlation analysis revealed SUC activity was significantly positively correlated with DOC, while oxidase activities were positively correlated with POC but significantly negatively correlated with Mg^2+^. Collectively, these results confirm that soil carbon turnover is fine-tuned by a “resource-microbe” regulatory network, specifically influenced by the availability of distinct carbon fractions and the soil ionic environment, rather than total organic carbon content. Slope gradients drive this process by altering resource distribution, thereby regulating the soil carbon cycle. For practical soil conservation: (1) Slopes <30°: adopt contour plowing, straw mulch, and organic fertilizer application to enhance soil enzyme activity and promote carbon sequestration. (2) Critical slopes (30°–45°): implement contour terraces and biochar application to improve soil conditions and reinforce carbon retention. (3) Slopes >45°: convert farmland to forest-grass vegetation to stabilize soil structure and halt carbon loss. Future research should focus on identifying key slope thresholds, regulating soil ionic environments, and managing vegetation inputs to halt progressive carbon cycle degradation.

## Data Availability

The datasets presented in this study can be found in online repositories. The names of the repository/repositories and accession number(s) can be found in the article/supplementary material.
